# Homology modeling and *in vivo* functional characterization of the zinc permeation pathway in a heavy metal P-type ATPase

**DOI:** 10.1093/jxb/ery353

**Published:** 2018-11-10

**Authors:** Gilles Lekeux, Jean-Marc Crowet, Cécile Nouet, Marine Joris, Alice Jadoul, Bernard Bosman, Monique Carnol, Patrick Motte, Laurence Lins, Moreno Galleni, Marc Hanikenne

**Affiliations:** 1 InBioS - Center for Protein Engineering (CIP), Biological Macromolecules, University of Liège, Liège, Belgium; 2 InBioS - PhytoSystems, Functional Genomics and Plant Molecular Imaging, University of Liège, Liège, Belgium; 3 Laboratory of Molecular Biophysics at Interfaces, Gembloux Agro-Bio Tech, University of Liège, Gembloux, Belgium; 4 InBioS - PhytoSystems, Laboratory of Plant and Microbial Ecology, Department of Biology, Ecology, Evolution, University of Liège, Liège, Belgium

**Keywords:** Arabidopsis, HMA4, homology modeling, *in vivo* imaging, metal transport, molecular dynamics, P-type ATPase, zinc

## Abstract

The P_1B_ ATPase heavy metal ATPase 4 (HMA4) is responsible for zinc and cadmium translocation from roots to shoots in *Arabidopsis thaliana*. It couples ATP hydrolysis to cytosolic domain movements, enabling metal transport across the membrane. The detailed mechanism of metal permeation by HMA4 through the membrane remains elusive. Here, homology modeling of the HMA4 transmembrane region was conducted based on the crystal structure of a ZntA bacterial homolog. The analysis highlighted amino acids forming a metal permeation pathway, whose importance was subsequently investigated functionally through mutagenesis and complementation experiments in plants. Although the zinc pathway displayed overall conservation among the two proteins, significant differences were observed, especially in the entrance area with altered electronegativity and the presence of a ionic interaction/hydrogen bond network. The analysis also newly identified amino acids whose mutation results in total or partial loss of the protein function. In addition, comparison of zinc and cadmium accumulation in shoots of *A. thaliana* complemented lines revealed a number of HMA4 mutants exhibiting different abilities in zinc and cadmium translocation. These observations could be instrumental to design low cadmium-accumulating crops, hence decreasing human cadmium exposure.

## Teaser

Three-dimensional modeling of the HMA4 protein in Arabidopsis reveals the zinc permeation pathway across the plasma membrane, and mutations in the pathway alter zinc and cadmium transport differently in plants.

## Introduction

P_IB_ ATPases, a subfamily of P-type ATPases, are metal cation transporters essential for the survival of organisms ranging from bacteria to eukaryotes such as plants and animals (Williams and Mills, 2005; Argüello *et al.*, 2011; Rosenzweig and Argüello, 2012). Some of these metals (e.g. zinc and copper) are fundamental for proper cell functioning (Hänsch and Mendel, 2009). These metals are required for about one-third of proteins with known structures where they act as enzymatic cofactors, structural stabilizers, or redox-active centers (Finney and O’Halloran, 2003; Andreini *et al.*, 2008). Other metals (e.g. cadmium and lead), as well as those aforementioned at high concentration, are toxic for cells (Goyer, 1997; Nzengue *et al.*, 2011; Clemens and Ma, 2016). Uptake, chelation, compartmentalization, and efflux mechanisms evolved to maintain metal homeostasis in cells, tissues, and organisms. P_IB_ ATPases are involved in metal efflux out to the periplasm in prokaryotes and out of the cell or into organelles in eukaryotes (Ma *et al.*, 2009; Palmer and Guerinot, 2009; Kambe *et al.*, 2015).

A combination of biochemical characterizations and phylogenetic studies allowed the classifation of P_IB_ ATPases in at least four subgroups having different substrate specificities and evolutive origins (Argüello, 2003; Thever and Saier, 2009; Chan *et al.*, 2010; Pedersen *et al.*, 2012; Hanikenne and Baurain, 2013; Smith *et al.*, 2014). Pumps from the P_IB-1_ subgroup are monovalent cations (e.g. Cu^+^ and Ag^+^) transporters. This subgroup is found in all domains of life and includes the well-characterized CopA bacterial transporter, as well as the human ATP7A and ATP7B proteins, whose mutations lead, respectively, to Menkes syndrome and Wilson’s disease (Odermatt *et al.*, 1993; Lutsenko and Petris, 2003). In contrast, P_IB-2_ ATPases transport divalent cations (e.g. Zn^2+^ and Cd^2+^) and are only present in bacteria and plants, with, for instance, the bacterial pumps ZntA and CadA and the *Arabidopsis thaliana* heavy metal ATPases 2–4 (HMA2–HMA4) (Nucifora *et al.*, 1989; Rensing *et al.*, 1997; Hussain *et al.*, 2004; Morel *et al.*, 2009).

The *A. thaliana* HMA2 and HMA4 transporters are key for zinc homeostasis, and an *hma2hma4* double mutant has a stunted growth phenotype, resulting from severe zinc deficiency in shoots (Mills *et al.*, 2003; Hussain *et al.*, 2004; Verret *et al.*, 2004). Together with its paralog HMA2, the HMA4 transporter is localized at the plasma membrane and is expressed in the root pericycle and in shoot cells bordering the xylem (vascular tissue) (Hussain *et al.*, 2004; Verret *et al.*, 2004; Sinclair *et al.*, 2007; Siemianowski *et al.*, 2013). HMA2 and HMA4 are responsible for zinc and cadmium translocation from roots to shoots (Hussain *et al.*, 2004; Wong and Cobbett, 2009; Cun *et al.*, 2014) and for zinc loading in seeds (Olsen *et al.*, 2016). Furthermore, high expression of HMA4 plays a major role in zinc and cadmium hyperaccumulation and hypertolerance in the Brassicaceae *Arabidopsis halleri* (Talke *et al.*, 2006; Courbot *et al.*, 2007; Hanikenne *et al.*, 2008, 2013) and *Noccaea caerulescens* (Ó Lochlainn *et al.*, 2011; Craciun *et al.*, 2012), a naturally selected extreme trait allowing them to colonize metal-polluted soils (Krämer, 2010; Hanikenne and Nouet, 2011).

P_IB_ ATPases have 6–8 transmembrane helices (TMs) forming the TM domain, two cytoplasmic catalytic domains, the actuator domain (A domain), and the ATP-binding domain (ATP domain) divided into a nucleotide-binding domain (N domain) and a phosphorylation domain (P domain), and for most of them N- and occasionally C-terminal cytosolic extensions. As members of the P-type ATPase superfamily, these proteins use the energy of ATP to transport their substrate following the E1/E2 Post–Albers cycle (Albers *et al.*, 1963; Post and Sen, 1965). During this cycle, the phosphorylation and dephosphorylation of an invariant Asp residue located in the P domain, as well as ion binding in the TM domain, trigger conformational changes allowing ion transport across the membrane (Kühlbrandt, 2004; Palmgren and Nissen, 2011; Rosenzweig and Argüello, 2012; Sitsel *et al.*, 2015).

N- and C-terminal cytosolic extensions of P_IB_ ATPases co-ordinate metals with high affinity thanks to metal-binding amino acids such as Cys, His, and Glu residues (Liu *et al.*, 2005; Eren *et al.*, 2006, 2007; Zimmermann *et al.*, 2009; Laurent *et al.*, 2016). The N-terminal extensions of P_IB-1_ and bacterial P_IB-2_ transporters have metal-binding domains (MBDs) with a typical βαββαβ-fold structure containing a consensus CxxC motif binding one Cu^+^ or one Zn^2+^ ion, respectively (Argüello *et al.*, 2007; Rosenzweig and Argüello, 2012). These MBDs are thought to have distinct functions in different proteins or even different functions when present in tandem in the same protein (González-Guerrero and Argüello, 2008; Drees *et al.*, 2015). A regulatory role has been proposed for the ZntA N-terminal MBD of *Escherichia coli* whose truncation or CxxC motif mutation results in reduced activity without altering its function (Mitra and Sharma, 2001; Liu *et al.*, 2006). The ZntA N-terminal MBD may achieve its function through interaction with a docking platform positioned at the membrane interface of the TM domain (Wang *et al.*, 2014). In plant P_IB-2_ ATPases, the N-terminal domain also displays an MBD with the βαββαβ-fold. However, its Zn^2+^-binding site is a CCxxE motif. In HMA4, this non-canonical site binds one Zn^2+^ atom with high affinity, and this interaction is essential for the function of the protein *in planta* (Zimmermann *et al.*, 2009; Laurent *et al.*, 2016). Similarly, the deletion of the HMA2 N-terminal domain results in decreased ATPase activity and also impairs the function of the protein *in planta* (Eren *et al.*, 2007; Wong *et al.*, 2009).

So far the function of the cytosolic terminal extensions of the plant P_IB_ ATPases have been extensively investigated (Verret *et al.*, 2005; Eren *et al.*, 2006, 2007; Wong *et al.*, 2009; Zimmermann *et al.*, 2009; Baekgaard *et al.*, 2010; Mills *et al.*, 2010; Laurent *et al.*, 2016). On the other hand, the TM domain has not been examined in detail. The TM domain of all P_IB_ ATPases has six core helices (TM1–TM6) preceded by two additional helices in many of them (TMA and TMB). A characteristic sequence of three amino acids (CPx motif) in TM4 together with amino acids in TM5 and TM6 may determine ion selectivity (Argüello, 2003; Hanikenne and Baurain, 2013; Smith *et al.*, 2014). The TM domain of HMA4 displays the CPx motif conserved in TM4 of P_IB-1_ and P_IB-2_ ATPases, and the P_IB-2_ subgroup signature sequences N(x)_7_K and DxG(x)_7_N on TM5 and TM6, respectively (Argüello, 2003; Hanikenne and Baurain, 2013; Smith *et al.*, 2014). The crystal structures of the Post–Albers cycle phophoenzyme ground state (E2P) and dephosphorylation intermediate (E2-P_i_) of the *Shigella sonnei* ZntA protein, a homolog of HMA4, has recently been determined (Wang *et al.*, 2014). This study proposed a mechanism of Zn^2+^ transport in which the TMs of ZntA form a permeation pathway across the membrane. This pathway starts with an electronegative funnel (E184, E214, and D348) that directs free Zn^2+^ ions to the intramembrane Zn^2+^-binding site. This site consists of C392 and C394 of the conserved CPx motif and D714 of the DxG(x)_7_N P_IB-2_ signature sequence. Their involvement in Zn^2+^ binding had already been characterized biochemically (Dutta et al., 2006, 2007; Liu *et al.*, 2006; Raimunda *et al.*, 2012). This site is capped by M187 and F210 to avoid Zn^2+^ backflow after binding. Extracellular Zn^2+^ release is then stimulated by E202 that is part of an exit pathway opened in the E2P state. In the E2-P_i_ intermediate state, the pathway closes; K693 [N(x)_7_K motif] located near D714 forms a salt bridge with the latter, preventing Zn^2+^ reflux to the binding site (Wang *et al.*, 2014). The importance of some of those residues for the ATPase activity and the function of the protein in bacteria has been tested (Dutta *et al.*, 2006, 2007; Okkeri and Haltia, 2006; Wang *et al.*, 2014; Zhitnitsky and Lewinson, 2014).

The level of sequence conservation between ZntA and HMA4 TM regions made the HMA4 TM domain amenable to structural homology modeling. It allowed examination of the conservation of transport mechanisms across bacterial and plant transporters, as well as putative plant-specific sequence features of the domain. The amino acids highlighted as important for transport in the model were then functionally characterized in plants. This complementary *in silico*/*in vivo* analysis allowed further advancement of our understanding of the HMA4-mediated mechanism of metal transport across the membrane in plants.

## Materials and methods

### Plant material, growth condition, and transformation

*Arabidopsis thaliana hma2hma4* double mutant plants (Col-0 background) (Hussain *et al.*, 2004) were used in all experiments. Prior to transformation, plants were grown on soil supplied with 1 mM ZnSO_4_ in a short-day growth chamber (22 °C and 8 h d^–1^ photoperiod) during 7 weeks. They were then transferred in long days (16 h d^–1^ photoperiod) where they were supplied with 3 mM ZnSO_4_ for 5 weeks to allow flowering. The plants were transformed using *Agrobacterium tumefaciens* by floral dipping (Clough and Bent, 1998).

For phenotyping on soil and metal accumulation analysis, first-generation (T_1_) heterozygous transgenic seeds (thus potentially including multiple insertion lines) were germinated on hygromycin B (20 µg ml^–1^) 1/2 MS (Murashige and Skoog) agar medium containing 1% sucrose in short days. After 14 d, seedlings were transferred to soil (potting mix, Brill TYPical, Tonerde 1/100 l), watered with tap water, and grown for 5 weeks in long days prior to imaging and sample harvesting.

### Cloning

To generate the *pAhHMA4-2::AtHMA4::GFP* cassette, the *A. thaliana HMA4* (*AtHMA4*) fragment was cloned into the *pAhHMA4-2::AhHMA4::GFP* pBluescript II KS+ vector (Nouet *et al.*, 2015) to replace the *AhHMA4* coding sequence, using the In-Fusion HD cloning kit (Takara). Site-directed mutagenesis, using the QuickChange Site-Directed Mutagenesis method (Agilent Technologies) and mutagenic primers (see Supplementary Table S1 at *JXB* online), was then performed on the newly constructed vector to create the TM variants (Table 1). The wild type (WT) and variant *pAhHMA4-2::AtHMA4::GFP* cassettes were finally cloned in a promoter-less variant of the pMDC32 vector (Curtis and Grossniklaus, 2003; Hanikenne *et al.*, 2008) after *Asc*I/*Pac*I excision from the pBluescript II KS+ vector.

**Table 1.** HMA4 amino acids involved in the TM Zn^2+^ transport pathway identified through 3D modeling and their corresponding mutants

Putative function	HMA4 WT	HMA4 mutants
Inlet funnel	N151-D181-D313	N151A D181A D313A N151AD181AD313A
Ionic interaction/hydrogen bond network	K138-R147-D149-N151- D181-E184-R310-D313	K138A D149A N151A D181A D313A N151AD181AD313A
Inlet gate	V154-F177	V154A V154S F177A F177L V154AF177A
Zn^2+^-binding site	C357-C359-D688	D688A
Zn^2+^-binding motif positioning	G356-G360-P366	G360A G356AG360A P366L
Zn^2+^ release	E169	E169A
Outlet gate	K667	K667A

### Metal accumulation analyses

Shoot tissues were cleaned with milliQ water and dried at 60 °C for 3 d. Shoot samples (10–50 mg of tissues) were then acid-digested in DigiPrep tubes with 3 ml of ≥65% HNO_3_ (Sigma-Aldrich) on a DigiPrep Graphite Block Digestion System (SCP Science) as follows: 15 min at 45 °C, 15 min at 65 °C, and 90 min at 105 °C. After cooling, sample volumes were adjusted to 10 ml with milliQ water, and 200 µl of ≥65% HNO_3_ was added. Metal concentrations were determined using inductively coupled plasma atomic emission spectroscopy (ICP-AES) with a Vista-AX instrument (Varian, Melbourne, Australia) as described (Nouet *et al.*, 2015).

### Confocal imaging

T_1_ seeds of *A. thaliana hma2hma4* plants expressing the WT and variant AtHMA4 proteins fused to green fluorescent protein (GFP) as described above were germinated on hygromycin B (20 µg ml^–1^) 1/2 MS agar medium containing 1% sucrose in short days. After 14 d, seedlings were transferred on the same medium without antibiotic. After 3 d, roots of 2–4 independent lines per construct from two independent experiments were analyzed. Images were collected at a 1024 × 1024 pixel resolution using a TCS SP5 inverted confocal laser microscope (Leica Microsystems) with a ×63 water immersion PlanApochromat 1.20 objective (Leica Microsystems) as previously described (Rausin *et al.*, 2010). An argon/ion laser (488 nm) was used for GFP excitation, and the emission light was dispersed and recorded between 500 nm and 540 nm. Within one experiment, all images were acquired with the same excitation and detection settings (PMT gain, offset,...) for all genotypes, with a PMT gain ensuring detection of GFP fluorescence only and excluding autofluorescence. To estimate HMA4 protein expression levels in root cells (Tillemans *et al.*, 2005; Dubeaux *et al.*, 2018), GFP fluorescence intensities were measured from confocal microscope images using ImageJ (https://imagej.nih.gov/ij/) and plot profile analysis. Briefly, in pericycle cells expressing HMA4 fused to GFP, 10 optical sections were drawn across the transversal plasma membranes. GFP fluorescence intensity values (*n*=20) were then used to calculate a mean fluorescence intensity for each independent mutant line.

### Homology modeling

The Uniprot code of the AtHMA4 protein is O64474 (http://www.uniprot.org). This protein has an N-terminal MBD (residues 1–96) that has been resolved by NMR (PDB ID: 2KKH) (Zimmermann *et al.*, 2009). In contrast, there is no known structure for the AtHMA4 TM domain (residues 97–702) and the C-terminal extension (residues 702–1172). A search for template models for the TM domain with the protein NCBI blast tool (https://blast.ncbi.nlm.nih.gov) (Johnson *et al.*, 2008) on the PDB (http://www.rcsb.org/pdb) (Berman *et al.*, 2000) yielded six results with sequence identity >30%: (i) 3J08 and 3J09 are CopA Cu^+^-transporting ATPases which have been reconstructed from electron microscopy with a resolution of 10 Å (Allen *et al.*, 2011); (ii) 3RFU and 4BBJ correspond to the crystal structures of CopA Cu^+^-transporting ATPases in E2-P_i_ and E2P states and with a resolution of 3.2 Å and 2.75 Å, respectively (Gourdon *et al.*, 2011; Andersson *et al.*, 2014); and (iii) 4UMV and 4UMW correspond to the crystal structures of the E2P and E2-P_i_ states of the ZntA Zn^2+^-transporting P_1B_ ATPase with 3.2 Å and 2.70 Å resolution, respectively (Wang *et al.*, 2014). Being the most similar to and sharing substrate specificity with AtHMA4, these last two structures were used as models for the homology modeling of the AtHMA4 protein. In addition, a multiple sequence alignment of these sequences and seven other plant or bacterial Cu- or Zn/Cd-transporting ATPases (Uniprot ID: Q5ZWR1, Q9SZW4, P0CW78, P0CW77, Q6GIX1, Q60048, and P30336) was obtained with Tcoffee (Notredame *et al.*, 2000) and also used to build the model with Modeller 9.14 software (Eswar *et al.*, 2007). The models were then submitted to GalaxyWeb for refinement (http://galaxy.seoklab.org/) (Ko *et al.*, 2012). The quality of the model was verified using the Swiss model server (https://swissmodel.expasy.org) through the QMEAN6 *Z*-score (Benkert *et al.*, 2008) and Procheck (Laskowski *et al.*, 1993). The electrostatic surface representation was made using the Adaptive Poisson Boltzmann Equation (APBS) plugin in PyMOL (Unni *et al.*, 2011).

### Molecular dynamics

The models were then used for molecular dynamic simulations with the Gromacs v4.5.4 software (Hess *et al.*, 2008). As HMA4 is a membrane protein, coarse-grained simulations have been carried out for protein insertion and building of the lipid membrane. Models were converted to a CG representation suitable for the MARTINI 2.1 forcefield (Marrink *et al.*, 2007) with the Martinize script and the coarse-grained protein was placed in a PLPC (1-palmitoyl,2-linoleyl-*sn*-glycero-3-phosphocholine) bilayer of 347 lipids with the insane tool (Wassenaar *et al.*, 2015). Water particles were then added, as well as ions to neutralize the system. A 5000 step steepest-descent energy minimization was performed to remove any steric clashes. An equilibration of 10 ns with protein under position restraint and a 20 fs time step has been carried on. Temperature and pressure were coupled at 300 K and 1 bar using the weak coupling Berendsen algorithm (Berendsen *et al.*, 1984) with τT=1 ps and τP=1 ps. Pressure was coupled semi-isotropically in *XY* and *Z*. Non-bonded interactions were computed up to 1.2 nm with the shift method. Electrostatics were treated with dielectric permittivity constant ε=15. The compressibility was 10^–5^ (1/bar). The system was then transformed to an atomistic resolution with backwards (Wassenaar *et al.*, 2014) and the protein replaced by the initial model. Atomistic simulations have been performed with the GROMOS96 54a7 force field (Poger and Mark, 2010; Poger *et al.*, 2010; Schmid *et al.*, 2011). All the systems studied were first minimized by steepest descent for 2000 steps. Then NVT and NPT equilibrations were carried on for 0.1 ns and 1 ns. The protein was under position restraints, and periodic boundary conditions (PBCs) were used with a 2 fs time step. Production runs were performed for 50 ns. All the systems were solvated with SPC water (Berendsen *et al.*, 1981) and the dynamics were carried out in the NPT conditions (300 K and 1 bar). Temperature was maintained by using the Nose–Hoover method (Nosé, 1984) with τT=0.5 ps, and a semi-isotropic pressure was maintained by using the Parrinello–Rahman barostat (Parrinello and Rahman, 1981) with a compressibility of 4.6 × 10^–5^ (1/bar) and τP=5 ps. Non-bonded interactions were evaluated using a twin-range cut-off scheme. Interactions within the shorter range cut-off (0.8 nm) were calculated every step, whereas interactions within the longer cut-off (1.4 nm) were updated every five steps, together with the pair list. In all the simulations, a reaction ﬁeld correction was applied to the electrostatic interactions beyond the long-range cut-off of 35 using a relative dielectric permittivity constant ε_RF_ of 62 (Tironi *et al.*, 1995). Bond lengths were maintained with the LINCS algorithm (Hess *et al.*, 1997). The trajectories were performed and analyzed with the GROMACS 4.5.4 tools as well as with homemade scripts and software, and 3D structures were analyzed with both PYMOL (DeLano Scientific, http://www.PyMOL.org) and VMD software (Humphrey *et al.*, 1996). The RMSD (root mean square deviation) is computed for the membrane domain along the simulations and represents the difference between two structures based on the distance between their respective atomistic co-ordinates.

## Results

### HMA4 3D model

**Fig. 1.**
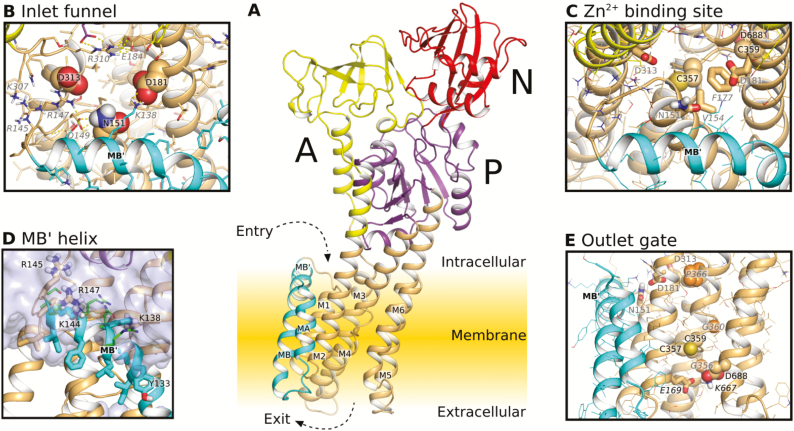
Homology model of HMA4 in E2P state. (A) View of the protein domains; transmembrane helices are colored in brown, except for helix MA, MB, and MB' colored in cyan, and the A, P, and N domains colored in yellow, purple, and red, respectively. (B) Close-up of the inlet funnel; key residues of the funnel and surrounding residues forming an ionic interaction/hydrogen bond network are named in black and italic gray, respectively. (C) Close-up of the Zn^2+^-binding site; key residues of the binding site, the inlet funnel, and the inlet gate are named in black, gray and italic gray, respectively. (D) Close-up of the MB' helix; the hydrophobic and hydrophilic residues of the MB' helix of the model are represented as sticks and the hydrophilic residues of the template structure 4UMV of *Shigella sonnei* Zn^2+^-ATPase are in green. Water is represented by a blue surface. (E) Close-up of the outlet gate; key residues of the binding site, the inlet funnel, associated with plasticity, and the outlet gate are named in black, gray, italic gray, and italic black, respectively.

The amino acid sequences corresponding to the TM regions of HMA4 and ZntA exhibit 33% sequence identity (Supplementary Fig. S1). Three-dimensional (3D) models of the *A. thaliana* HMA4 protein lacking its N- and C-terminal cytosolic domains were therefore obtained using as reference the ZntA crystal structures of the E2P and E2-P_i_ reaction cycle intermediates (Wang *et al.*, 2014). HMA4 and ZntA share similar overall structure, typical of P_IB_ ATPases, with eight transmembrane segments defining the TM domain, and intracellular A, P, and N domains (Fig. 1A). Both proteins are organized similarly in the E2P and E2-P_i_ steps and display a similar Zn^2+^ permeation pathway through the membrane. Identically to ZntA, HMA4 has a Zn^2+^-binding site made of C357, C359, and D688, with K667 close by, and capped by V154 and F177 (Fig. 1C, E). E169 is observed at the same position on the release pathway as E202 of ZntA (Fig. 1E).

**Fig. 2.**
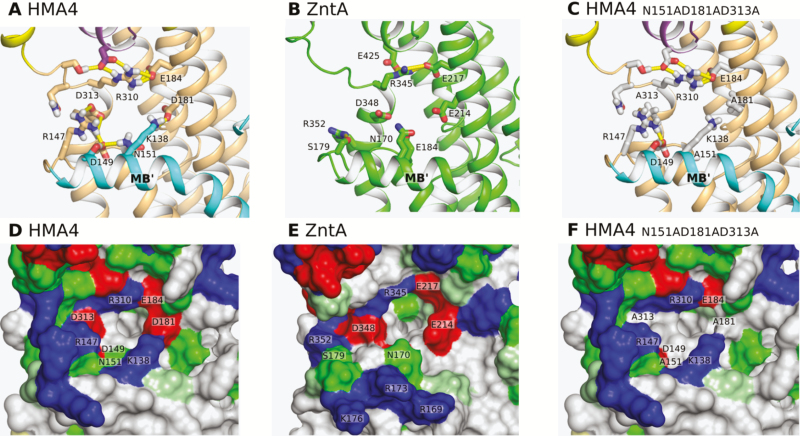
Ionic interaction/hydrogen bond network and distribution of residue types at the surface of the TM domain of HMA4, ZntA, and the NADADA triple mutant of HMA4. (A–C) Hydrogen bonds are represented as yellow sticks. (D–F) Hydrophobic, polar, positively charged, negatively charged, and small (Gly and Pro) residues are colored in white, green, blue, red, and pale green respectively.

In contrast, while both HMA4 and ZntA have a Pro residue downstream of the CPC motif, in positions P366 and P401, respectively, this motif is surrounded by two Gly residues (G356 and G360) in HMA4 instead of one in ZntA (G391) (Fig. 1E; Supplementary Fig. S1). Additional differences between the two proteins lie in the entrance area. The inlet funnel of HMA4 constituted of N151, D181, and D313 is less electronegative compared with ZntA and is part of an ionic interaction/hydrogen bond network surrounding the funnel (K138, R147, D149, N151, D181, E184, R310, and D313) (Figs 1B, 2A, B, D, E). This network is present to a lesser extent in ZntA (E217, R345, and E425) (Fig. 2B). Furthermore, both proteins exhibit an amphipathic helix (MB') at the intracellular membrane interface (Figs 1D, 2). A clear amphipathic partition is visible between hydrophobic and hydrophilic residues along the helix and at the water–lipid interface (Fig. 1D). However, while the positive R169, R173, and K176 residues of the ZntA amphipathic helix are exposed to the cytoplasm, the K138 and R147 residues of HMA4 are turned towards the funnel and involved in the ionic interaction/hydrogen bond network (Figs 1B, D, 2A, B, D, E).

### *In vivo* functional assay

To examine *in planta* the functional importance of the amino acids depicted in the model, HMA4 transmembrane variants were generated by directed mutagenesis (Table 1). In most cases, single amino acids were mutated to an Ala residue. A triple mutant N151AD181AD131A (NADADA) and two double mutants V154AF177A (VAFA) and G356AG360A (GAGA) were also generated. P366 was replaced by a Leu residue instead of an Ala residue to evaluate the importance of its conformational rigidity. The V154 and F177 residues were mutated to Ala residues but also to Ser and Leu residues, respectively, to test their hydrophobic and spatial properties. The native *HMA4* (WT) and mutated genes were fused to the *GFP* gene (*HMA4::GFP*) and expressed under the control of the endogenous *A. halleri HMA4* promoter 2 (*pAhHMA4-2*) in the loss-of-function *hma2hma4 A. thaliana* mutant (Hussain *et al.*, 2004; Hanikenne *et al.*, 2008; Nouet *et al.*, 2015). *pAhHMA4-2* was selected as, in contrast to the *A. thaliana HMA4* promoter, it supports an expression level sufficient to enable good complementation of the mutant phenotype (Nouet *et al.*, 2015; Laurent *et al.*, 2016). As previously shown (Nouet *et al.*, 2015), GFP fusion does not abolish HMA4 function and allows a simultaneous protein expression and localization analysis *in planta*.

**Fig. 3.**
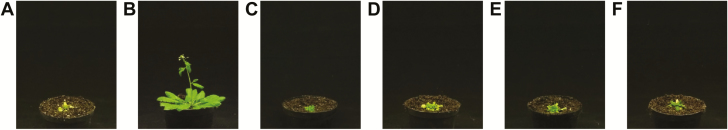
Complementation of the *A. thaliana hma2hma4* zinc deficiency phenotype. D688A (C), P366L (D), E169A (E), and K667A (F) HMA4 variants fused to GFP were expressed in *hma2hma4* plants under the control of the *pAhHMA4-2* promoter. The plant phenotypes are shown after 5 weeks of growth on soil without zinc supplementation. *hma2hma4* plants not transformed (A) or expressing the native HMA4 fused to GFP (B) were used as negative and positive controls, respectively. Pictures are representative of the observations of at least 12 independent lines/plants from two independent experiments for each genotype.

On soil watered with tap water, the *hma2hma4* mutant plant showed its typical zinc deficiency phenotype (Fig. 3A) (Hussain *et al.*, 2004). Expression of the D688A, P366L, E169A, and K677A HMA4 variants failed in rescuing this deficiency. Indeed, plants expressing these variants had the same stunted growth and chlorotic aspect as the *hma2hma4* mutant, suggesting that these variants are non-functional (Fig. 3C–F). In contrast, expression of the native HMA4 protein as well as all the remaining variants complemented the *hma2hma4* phenotype and allowed the plant to develop normally until seed setting (Fig. 3B; Supplementary Fig. S2).

### Protein localization, expression, and stability

**Fig. 4.**
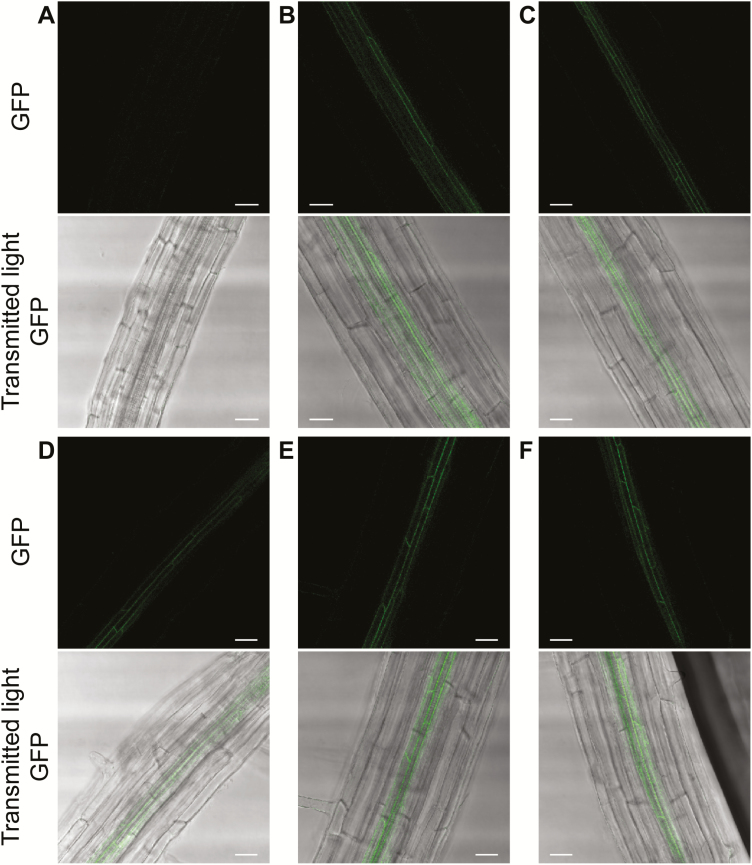
HMA4 TM variant localization in *A. thaliana*. GFP fusions of D688A (C), P366L (D), E169A (E), and K667A (F) HMA4 variants were imaged by confocal microscopy in roots of 18-day-old T_1_ seedlings. The variants are expressed in the *A. thaliana hma2hma4* mutant under the control of the *pAhHMA4-2* promoter. *hma2hma4* seedlings either not transformed (A) or expressing the native HMA4 fused to GFP (B) were used as negative and positive controls, respectively. The images are, for each genotype, representative of 2–4 independent lines from two independent experiments. Scale bars*=*25 µm.

The D688A, P336L, E169A, and K677A HMA4 variants may have lost the ability to complement the *hma2hma4* phenotype because of a mislocalization of the protein or an absence of expression. Indeed, mutations in the TM domain of HMA4 may result in altered intracellular localization and/or protein stability. Therefore, *hma2hma4* roots of seedlings expressing the TM domain variants fused to GFP were examined by confocal microscopy. As shown previously (Nouet *et al.* 2015), the native protein localized in the plasma membrane of pericycle cells of the root (Fig. 4B). The four non-functional variants (Fig. 4C–F), as well as all other variants (Supplementary Fig. S3), were similarly detected in the plasma membrane of pericycle cells. No GFP aggregation was observed in cells of all the lines analyzed. As expected, non-transformed *hma2hma4* seedlings were not emitting any fluorescence (Fig. 4A). Moreover, protein expression levels in cells were estimated for all variants through quantification of GFP fluorescence. No significant differences were observed between variants, suggesting similar expression levels (Supplementary Fig. S4). To assess further the stability of these mutant proteins, molecular dynamic simulations were carried out during 25 ns for each non-functional mutant, and the RMSD was followed to estimate the structural fluctuations. Values <3 Å were observed, indicative of no alteration of stability (Supplementary Table S2). Altogether, the *in planta* confocal microscopy experiments and *in silico* simulations suggested that the proteins were expressed at similar levels, localized as expected, and were stable. Therefore, the inability of the D688A, P366L, E169A, and K667A HMA4 mutants to complement the *hma2hma4* phenotype most probably results from a loss of Zn^2+^ transport function of the proteins.

### Zinc and cadmium accumulation in plant shoot

**Fig. 5.**
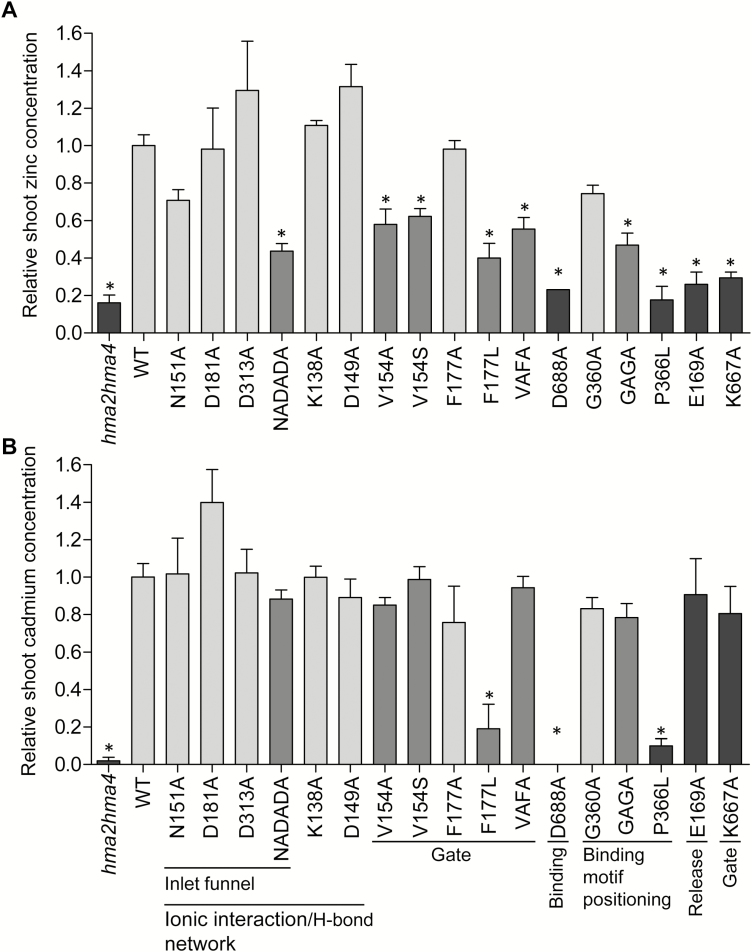
Zinc and cadmium accumulation in shoots of complemented plants. *hma2hma4* mutant and mutant plants expressing the native HMA4 protein (WT) or the TM variants were grown for 5 weeks on soil watered with tap water. Zinc (A) and cadmium (B) contents were measured in shoot tissues. Values relative to the WT are means ±SEM of 6–12 pools of two independent lines/plants from two independent experiments for each genotype. The data were analyzed with a one-way ANOVA test followed by Tukey’s multiple comparison tests. Statistically significant differences (*P*<0.05) from the WT mean are indicated by *. The dark gray, medium gray, and light gray colors correspond respectively to plants exhibiting the stunted growth *hma2hma4* phenotype, the WT phenotype but a significant decrease of zinc in shoots, and the WT phenotype with a zinc level similar to that of WT plants.

To support these observations further, zinc and cadmium accumulation in shoot were measured by ICP-AES in rosette leaves of 5-week-old *hma2hma4* and *hma2hma4* plants expressing the native HMA4 protein or the TM variants grown on soil watered with tap water. Plants expressing the native HMA4 protein (WT) accumulated ~5-fold higher zinc in shoots than the *hma2hma4* mutant, reflecting the inability of the latter to translocate zinc from root to shoot (Fig. 5A). The D688A, P336L, E169A, and K677A variant plants showed zinc shoot levels equal to those of *hma2hma4* plants, explaining their identical visual phenotype (Figs 3, A). As expected, N151A, D181A, D313A, K138A, D149A, F177A, and G360A variant lines accumulated zinc shoot concentrations similar to the WT. In contrast, a shoot zinc level intermediate between *hma2hma4* and WT plants was measured in the NADADA, V154A, V154S, F177L, VAFA, and GAGA variants (Fig. 5A), suggesting partially altered molecular function for these variants. Interestingly, mutations in a number of TM residues altered cadmium accumulation in shoot in a different way from that for zinc. Indeed, whereas the translocation of zinc to shoot was intermediately or strongly decreased in NADADA, V154A, V154S, VAFA, GAGA, E169A, and K667A variant-expressing plants, cadmium levels equivalent to native HMA4-expressing *hma2hma4* plants were measured (Fig. 5B). It is particularly striking that substitution of E169 and K667 by Ala residues results in a total loss of function of Zn^2+^ transport (Figs 3, 5A) but can still transport Cd^2+^ as the native protein (Fig. 5B). Finally, the F177L HMA4 variant was the only one triggering reduced shoot cadmium accumulation while retaining the ability to complement the *hma2hma4* phenotype (Fig. 5; Supplementary Fig. S2).

## Discussion

Through combined 3D modeling and *in vivo* functional characterization, new insights into the molecular mechanisms of the HMA4-mediated metal transport across the membrane are presented. Building a 3D model of HMA4 based on the ZntA crystallographic structure (Wang *et al.*, 2014) allowed the identification of amino acids of HMA4 forming a Zn^2+^ permeation pathway in the membrane that is mostly conserved compared with the structure of the ZntA protein (Wang *et al.*, 2014), with some peculiarities. Most, but not all, of these amino acids are highly conserved among plant P_IB-2_ ATPases (Supplementary Fig. S5), suggesting that our findings may also apply to HMA4 homologs in other plant species. The functional importance of a number of these amino acids, such as those highly conserved in the NX(7)KX(10,20)DXGX(7)N motif (K667 and D688) found in TM5 and TM6 (Argüello, 2003), was previously assessed in biochemical assays *in vitro* (Dutta *et al.*, 2006, 2007; Liu *et al.*, 2006; Okkeri and Haltia, 2006; Wang *et al.*, 2014) or *in vivo* (Dutta *et al.*, 2006; Zhitnitsky and Lewinson, 2014) using ZntA, and was here examined in detail using complementation experiments in plants.

In parallel, the localization, the expression level, and the stability of the HMA4 TM variants used in complementation experiments have been examined (Fig. 4; Supplementary Figs S3, S4; Supplementary Table S2). HMA4 localizes in the plasma membrane of plant tissues (Verret *et al.*, 2004; Courbot *et al.*, 2007; Siemianowski *et al.*, 2013; Nouet *et al.*, 2015). Mutations in the CCTSE motif of the HMA4 N-terminal domain and truncation of the HMA2 N-terminal domain do not alter their intracellular localization (Wong *et al.*, 2009; Laurent *et al.*, 2016). Here, all HMA4 TM variants were imaged in the plasma membrane of the pericycle cells in roots (Fig. 4; Supplementary Fig. S3). The signal targeting HMA4 to the plasma membrane in plants might therefore involve amino acid residues other than those involved in the Zn^2+^ pathway in the TM domain or be found in the cytoplasmic and C-terminal domains of the protein (Lefebvre *et al.*, 2004; Wong *et al.*, 2009). Together with the *in silico* dynamic molecular simulations (Supplementary Table S2), these localization experiments also attest to the stability and the similar expression of the TM variants (Fig. 4; Supplementary Figs S3, S4). Therefore, the inability of some HMA4 TM variants to complement the *hma2hma4* mutant can probably be attributed to a loss of metal transport activity.

The HMA4 Zn^2+^ permeation pathway is divided into three parts: the inlet funnel, the intramembrane Zn^2+^-binding site, and the release pathway. The entrance part of HMA4 is of particular interest as the amino acid configuration surrounding the inlet funnel is different compared with ZntA. The inlet funnel of HMA4 includes negatively charged D181 and D313 residues corresponding to the E214 and D348 residues of ZntA thought to be responsible for free Zn^2+^ attraction to the membrane-binding site thanks to their electronegativity (Wang *et al.*, 2014). In contrast, the third negatively charged residue (E184) of the inlet funnel of ZntA is replaced by the N151 residue in HMA4, decreasing the electronegativity of the funnel (Figs 1B, 2A, B, D, E). This change appears to be common among plant P_IB-2_ ATPases (Supplementary Fig. S5). Moreover, in HMA4, the K138, R147, D149, N151, D181, E184, R310, and D313 residues are organized in a ionic interaction/hydrogen bond network which might stabilize the structure and consolidate the entrance area (Hong, 2014). The three amino acids forming the funnel (N151, D181, and D313) are also part of this network (Figs 1B, 2A, B, D, E). The K138 and R147 of the network contribute to an alteration of the electronegative surface around the funnel of HMA4 compared with ZntA (Figs 1B, 2A, B, D, E). Those two residues are absent in ZntA and display important sequence variations among plant P_IB-2_ ATPases (Supplementary Fig. S5). Both consolidation of the entrance and changes of electronegativity of the funnel may alter zinc entrance into the permeation pathway of HMA4 compared with ZntA. To accommodate the presence of these positively charged amino acids, Zn^2+^ might be delivered to the funnel as Zn^2+^ ligands (e.g. nicotianamine, glutathione, and organic acids) (Sinclair and Krämer, 2012; Wang *et al.*, 2014).

In addition, K138 and R147 are positioned on MB' bordering the funnel in HMA4. In contrast, the corresponding residues of ZntA MB' are exposed to the cytoplasm (Figs 1B, D, 2A, B, D, E) (Wang *et al.*, 2014). In ZntA, this structure is thought to form a platform allowing interaction with the N-terminal MBD through electrostatic complementation and van der Waals interaction (Wang *et al.*, 2014). A similar mechanism of interaction between the CopA TM domain and a cellular Cu^+^ chaperone delivering the metal to the TM domain has also been proposed (González-Guerrero and Argüello, 2008; Gourdon *et al.*, 2011; Mattle *et al.*, 2013; Padilla-Benavides *et al.*, 2013). No such chaperone exists for Zn^2+^. In ZntA, this interaction is suggested to serve as an autoregulation process (Okkeri and Haltia, 2006; Dutta *et al.*, 2007; Wang *et al.*, 2014). The surroundings of the inlet funnel of HMA4, including MB' and the ionic interaction/hydrogen bond network, might be involved in interaction with the HMA4 N-terminal domain. As this platform is different from the corresponding region of ZntA, the interaction mechanism may also differ. These differences are shown in Supplementary Fig. S6 and Fig. 2D, E that present, respectively, the distribution of residue types at the surface of the MBDs and membrane domains of HMA4 and ZntA. For example, the negatively charged residues of the MDB of ZntA are thought to interact with the positively charged residues of MB' (Wang *et al.*, 2014). The same regions of HMA4 MBD and MB' correspond to two hydrophobic surfaces that are putatively compatible. Further work will be required to verify if such an interaction takes place *in vivo* in Arabidopsis. Morever, as the sequence of the MB' region appears more variable than other TMs among among plant P_IB-2_ ATPases (Supplementary Fig. S5), these interactions might contribute to fine-tuning of the protein activity.

In the complementation assays, none of the residues forming the entrance funnel and its vicinity taken separately was essential for the function of the HMA4 protein *in planta* (Fig. 5A; Supplementary Fig. S2). The loss of either a single negative or positive charge might not sufficiently alter the electronegative surface of the funnel and the ionic interaction/hydrogen bond network to trigger a dysfunction of the protein. In contrast, the combination of the three N151A, D181A, and D313A mutations significantly decreased the electronegativity of the funnel and altered the ionic interaction/hydrogen bond network, and as a result intermediately impaired Zn^2+^ transport to the shoot (Figs 2C, F, 5A).

In the HMA4 protein, the amino acids corresponding to the C392, C394, and D714 high-affinity intramembrane Zn^2+^-binding site of ZntA are the C357, C359, and D688 residues. The D688 residue of HMA4 is highly conserved in P_IB-2_ ATPases (Hanikenne and Baurain, 2013) and is essential for the function of the protein (Figs. 3, 5). Similarly, mutations of the corresponding Asp residue of ZntA inactivate the pump (Dutta *et al.*, 2006, 2007; Okkeri and Haltia, 2006; Wang *et al.*, 2014; Zhitnitsky and Lewinson, 2014). The two cysteine residues are part of the canonical CPx motif of P_IB_ ATPases and are required for the function of the protein (Argüello, 2003; Mills *et al.*, 2005; Williams and Mills, 2005). The HMA4 CPC motif is surrounded by two Gly residues (G356 and G360) that may be important for the plasticity of the Zn^2+^-binding site. It might require a higher flexibility compared with ZntA that instead has a single Gly residue (G391) bordering the motif (Fig. 1E; Supplementary Fig. S1). It is interesting to note that an ACPCA motif is much more frequent among plant P_IB-2_ ATPases than the GCPCG motif found in HMA4 (Supplementary Fig. S5). However, the Zn^2+^ transport to the shoot of plants expressing the HMA4 variant where the two Gly residues are mutated (GAGA variant, thus corresponding to the ACPCA motif) was significantly decreased, suggesting that HMA4 might be more Zn efficient than many of its plant homologs (Fig. 5A). This hypothesis could be tested with the functional analysis of an ACPCA/GCPCG mutant of the tobacco HMA4 for instance. Moreover, our analysis pointed out the importance of the P366 residue of HMA4 located downstream of the CPC motif. It might be important for the positioning of the motif in the protein core as its replacement by a Leu residue in the P366L variant completely disrupted Zn^2+^ transport *in planta* (Figs 1E, 3D, 5A). As part of a CPCx(4)SxP motif of TM4, this residue is highly conserved among plant P_IB-2_ ATPases (Supplementary Fig. S5).

The two hydrophobic residues preceding the intramembrane metal-binding site of HMA4 are V154 and F177, corresponding to M187 and F210 of ZntA (Wang *et al.*, 2014) (Fig. 1C; Supplementary Fig. S1). If the Phe residue is highly conserved among plant P_IB-2_ ATPases, the Val residue is more variable and appears to be the derived state compared with the Met residue found in ZntA (Supplementary Fig. S5). The Phe residues are in the same position as an N106 in H^+^ P-type ATPases proposed to act as a gatekeeper (Pedersen *et al.*, 2007; Ekberg *et al.*, 2013). Together, the V154 and F177 residues of HMA4 might fulfill this function, preventing Zn^2+^ release back outside upon entry. The hydrophobic character of V154 as well as its steric hindrance are functionally important as the V154 HMA4 variants only partially complemented the *hma2hma4* mutant (Fig. 5A). Surprisingly, the replacement of the F177 residue by an Ala residue had no effect on the protein (Fig. 5A) (Zhitnitsky and Lewinson, 2014), whereas its substitution by a Leu residue significantly impaired its function (Fig. 5A). The VAFA double mutant exhibited an intermediate Zn^2+^ transport capacity as well. These results suggest that V154 is able to assume the steric hindrance function preventing Zn^2+^ backflow alone. Yet the configuration of the residue in position 177 is important, as shown with the F177L mutant (Fig. 5).

Our results suggest that the extracellular Zn^2+^ release mechanism is similar between HMA4 and ZntA. As has already been observed with the SERCA pump, the release pathway is opened in the E2P state, and closed in the E2-P_i_ state (Olesen *et al.*, 2007; Toyoshima *et al.*, 2007). In this state, as also happens in the *A. thaliana* H^+^-ATPase AHA2, the K667 residue of HMA4 would interact with D688 to avoid Zn^2+^ backflow as well as counter ion passage (Pedersen *et al.*, 2007). This switch from an opened E2P state to a closed E2-P_i_ is illustrated in our molecular dynamic simulations. Indeed, in the E2P state, external water molecules were able to enter the putative exit channel up to the K667 and D688 residues while they were not in the subsequent E2-P_i_ state (Supplementary Fig. S7). Zn^2+^ exit might then be stimulated by the negatively charged E169 residue of HMA4, located at the same position on the outlet pathway as the E202 residue of ZntA (Fig. 1E) (Wang *et al.*, 2014). As K667A and E169A HMA4 variants are both totally inactive in plants, these two highly conserved amino acids are of major importance for the protein function *in vivo* (Figs 3, 5; Supplementary Fig. S5).

Our modeling and *in vivo* complementation assays suggest that the function of the TM domain in the transport mechanism is overall conserved among HMA4 and ZntA. However, detailed functional analysis also revealed significant differences, especially in the structure of the entrance funnel. Furthermore, if four mutations, associated with the intramembrane-binding site and with metal release, fully abolished the function of the HMA4 protein *in planta*, a number of variants displayed partial activity that was sufficient to complement the visual phenotype of the *hma2hma4* mutant, but resulted in decreased shoot zinc accumulation. These accessory residues, namely N151, D181, D313, V154, G356, and G360, might be required for optimal transit of Zn^2+^ through the membrane.

Interestingly, observation of zinc accumulation in shoots of plants expressing null or partially affected HMA4 variants revealed that the difference between the zinc level required in the shoot for normal development and the level leading to a stunted growth phenotype is very narrow (Figs 3, 5A; Supplementary Fig. S2). In our growth conditions, ~34 ppm zinc in the shoot appeared to be sufficient for normal development and completion of the life cycle in Arabidopsis, while ~19 ppm zinc were insufficient.

Finally, comparison of zinc and cadmium accumulation in the shoot of *A. thaliana* complemented lines expressing the different HMA4 variants produced interesting observations. Whereas some variants were completely or partially unable to transport Zn^2+^, their ability to transport Cd^2+^ was similar to that of the native protein when expressed in *hma2hma4* plants (Fig. 5). Interpreting these peculiar data is not trivial, as the Zn^2+^ and Cd^2+^ cations have very similar properties. The differential effect of the F177L mutation on zinc and cadmium accumulation in plant shoots is particularly interesting. Indeed, in plants expressing this variant, the cadmium level in the shoot was significantly decreased while the zinc concentration, although also reduced, remained sufficient for normal growth of Arabidopsis (Fig. 5; Supplementary Fig. S2). This is in contrast to several attempts aimed at modifying HMA4-related proteins to reduce cadmium accumulation in tobacco leaves that resulted in drastically altered zinc homeostasis and consequently strongly impaired growth (Liedschulte *et al.*, 2017). The F177 amino acid of HMA4, as well as the majority of the important amino acids of the Zn^2+^ pathway described above, are conserved in *Nicotiana tabacum* HMA4 (NtHMA4) and *Oryza sativa* HMA2 (OsHMA2) and HMA3 (OsHMA3) proteins (Supplementary Fig. S8). NtHMA4 and OsHMA2 are responsible for zinc and cadmium root to shoot translocation in tobacco and rice, respectively (Satoh-Nagasawa *et al.*, 2012; Takahashi *et al.*, 2012; Yamaji *et al.*, 2013; Hermand *et al.*, 2014; Liedschulte *et al.*, 2017). OsHMA3 contributes to root tonoplast cadmium sequestration, thus lowering root to shoot Cd translocation when active (Ueno *et al.*, 2010; Miyadate *et al.*, 2011; Sasaki *et al.*, 2014; Yan *et al.*, 2016). Therefore, mutating the Phe residue corresponding to the *A. thaliana* HMA4 F177 in NtHMA4 and OsHMA2 proteins might decrease their shoot cadmium level while maintaining sufficient zinc translocation to the shoot to support their development. Considering that a trade-off between slightly altered zinc content and strongly reduced cadmium accumulation is acceptable, this might represent a new strategy to decrease human cadmium exposure through tobacco leaves and rice seeds. Similarly, expressing in rice an OsHMA3 variant with mutations in the residues corresponding to the *A. thaliana* HMA4 E169 or K667 would ensure unaltered cadmium vacuolar sequestration in roots but, through reduced zinc sequestration in roots, allow increased zinc accumulation in shoot for biofortification.

## Supplementary data

Supplementary data are available at *JXB* online.

**Table S1.** Mutagenic primers.

**Table S2.** RMSD (in nm) for molecular dynamic simulations.

**Fig. S1.** Amino acid sequence alignment of *Shigella sonnei* ZntA and *Arabidopsis thaliana* HMA4.

**Fig. S2.** Complementation of the *A. thaliana hma2hma4* zinc deficiency phenotype.

**Fig. S3.** HMA4 TM variant localization in *A. thaliana*.

**Fig. S4.** HMA4 TM variant expression level in *A. thaliana*.

**Fig. S5.** Sequence conservation of the TM region among plant P_IB-2_ ATPases.

**Fig. S6.** Distribution of residue types at the surface of the N-terminal MBDs of HMA4 and ZntA.

**Fig. S7.** View of the transmembrane domain of HMA4 in the E2P and E2-P_i_ states in a membrane environment during molecular dynamic simulations.

**Fig. S8.** Amino acid sequence alignment of *Arabidopsis thaliana* HMA4, *Nicotiana tabacum* HMA4, *Oryza sativa* HMA2, and *Oryza sativa* HMA3.

Supplementary Figures S1-S8 and Tables S1-S2

## Abbreviations:

A domain: actuator domain
3D: three-dimensional
MBD: metal-binding domain
N domain: nucleotide-binding domain
P domain: phosphorylation domain
TM: transmembrane helix
