# Supplementary material for: Homology modeling and in vivo functional characterization of the zinc permeation pathway in a heavy metal P-type ATPase
Source: J Exp Bot. 2018 Nov 10;70(1):329–41. doi: 10.1093/jxb/ery353 (PMC6305203; doi:10.1093/jxb/ery353)
Supplement: Supplementary Figures S1-S8 and Tables S1-S2 [file ery353_suppl_supplementary_figures_s1-s8_tables_s1-s2.pdf]

```
ZntA      LKENLPLITLIVMMAISWGLEQFNHPFGQLAF--IATTLVGLYPIARQALRLIKSGSYFA 182
HMA4      FKNKWPSPFAV-VSGLLLLLLSFLKFVYSPLRWLAVAAVAAGIYPILAKAFASIKRPR-ID 149

ZntA      IETLMSVAAIGALFIGATAEAAMVLLLFLIGERLEGWAASRARQGVSALMALKPETATRL 242
HMA4      INILVIITVIATLAMQDFMEAAAVVFLFTISDWLETRASYKATSVMQSLMSLAPQKAIIA 209

ZntA      RNGEREEVAINSLRPGDVIEVAAGGRLPADGKLLSPFASFDESALTGESIPVERATGDKV 302
HMA4      ETG--EEVEVDEVKVDTVVAVKAGETIPIDGIVVDGNCEVDEKTLTGEAFPVPKQRDSTV 267

ZntA      PAGATSVDRLVTLEVLSEPGASAIDRILKLIEEAEERRAPIERFIDRFSRIYTPAIMAVA 362
HMA4      WAGTINLNGYICVKTTSLAGDCVVAKMAKLVEEAQSSKTKSQRLIDKCSQYYTPAIILVS 327

ZntA      LLVTLVPPLLFAASWQEWIYKGLTLLLIGCPCALVISTPAAITSGLAAAARRGALIKGGA 422
HMA4      ACVAIVPVIMKVHNLKHWFHLALVVLVSGCPCGLILSTPVATFCALTKAATSGLLIKSAD 387

ZntA      ALEQLGRVTQVAFDKTGTLTVGKPRVTAIHPA-TGISESELLTLAAAVEQGATHPLAQAI 481
HMA4      YLDTLSKIKIVAFDKTGTITRGEFIVIDFKSLSRDINLRSLLYWVSSVESKSSHPMAATI 447

ZntA      VREAQVAELAI--PTAESQRALVGSGIEAQVNGERVLICAAGKHPADAF--AGLINELES 537
HMA4      VDYAKSVSVEPRPEEVEDYQNFPGEGIYGKIDGNDIFIGNKKIASRAGCSTVPEIEVDTK 507

ZntA      AGQTVVLVVRNDDVLGIIALQDTLRADAATAISELNALGVKGVILTGDNPRAAAAIAGEL 597
HMA4      GGKTVGYVYVGERLAGFFNLSDACRSGVSQAMAELKSLGIKTAMLTGDNQAAAMHAQEQL 567

ZntA      GL---EFKAGLLPEDKVKAVTKLNQHAPLAMVGDGINDAPAMKAAAIGIAMGSG-TDVAL 653
HMA4      GNVLDVVHGDLLPEDKSRIIQEFKKEGPTAMVGDGVNDAPALATADIGISMGISGSALAT 627

ZntA      ETADAALTHNHLRGLVQMIELARATHANIRQNITIALGLKGIFLVTTLLGMTGLWLAVLA 713
HMA4      QTGNIILMSNDIRRIPQAVKLARRARRKVVENVCLSIILKAGILALAFAGHPLIWAAVLV 687

ZntA      DTGATVLVTANALRLLRRR 732
HMA4      DVGTCLLVIFNSMLLLREK 706
```

Figure S1. Amino acid sequence alignment of *Shigella sonnei* ZntA and *Arabidopsis thaliana* HMA4. The HMA4 amino acids depicted by the 3D model as important for Zn^{2+} transport are highlighted. The residues forming the ionic interaction/hydrogen bond network are highlighted in cyan, with the inlet funnel amino acids underlined. The residues involved in Zn^{2+} binding and responsible for the binding motif positioning are respectively highlighted in red and gray. The amino acids forming the inlet and outlet gates are respectively highlighted in green and pink. The residue involved in Zn^{2+} release is highlighted in yellow. The alignment was obtained using Clustal Omega with default settings (Sievers et *al*., 2011. Molecular systems biology **7**, 539).

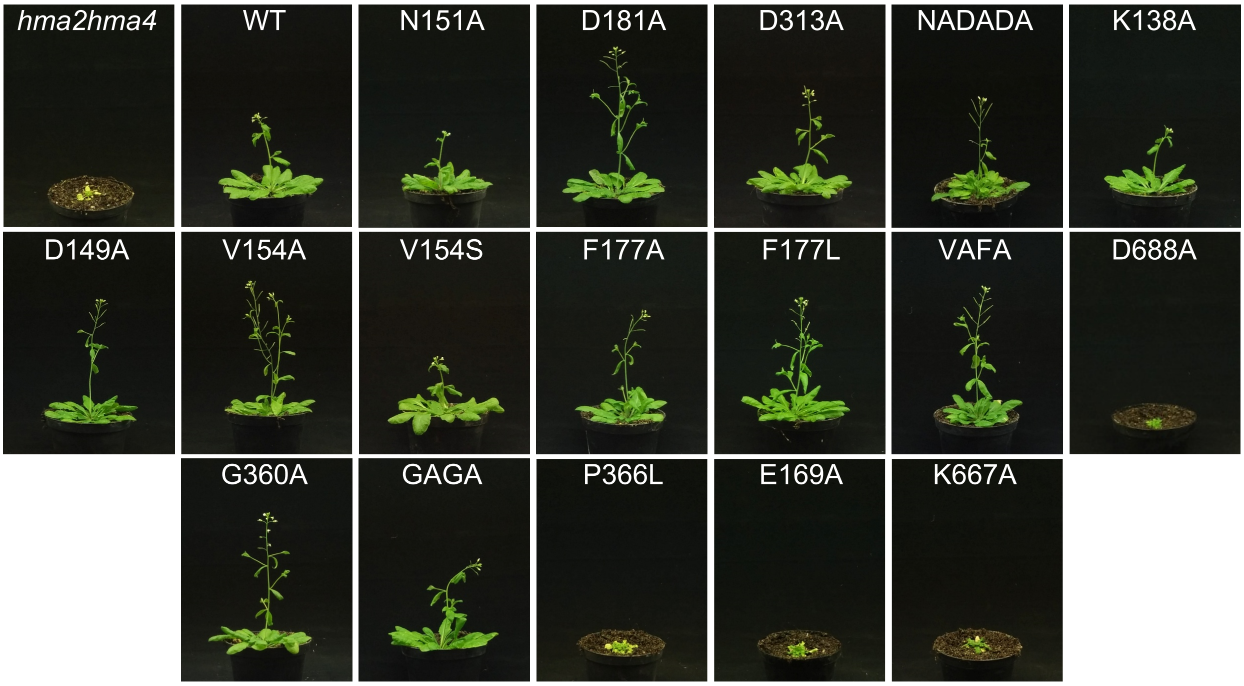

Figure S2. Complementation of the *A. thaliana hma2hma4* zinc deficiency phenotype. HMA4 variants fused to the GFP protein were expressed in *A. thaliana hma2hma4* plants under the control of the *pAhHMA4-2* promoter. The plant phenotypes are shown after 5 weeks of growth on standard soil without zinc supplementation. *hma2hma4* plants non transformed and expressing the native HMA4 fused to GFP (WT) were respectively used as negative and positive controls. Pictures are representative of the observations of at least 12 independent lines/plants from two independent experiments for each genotype.

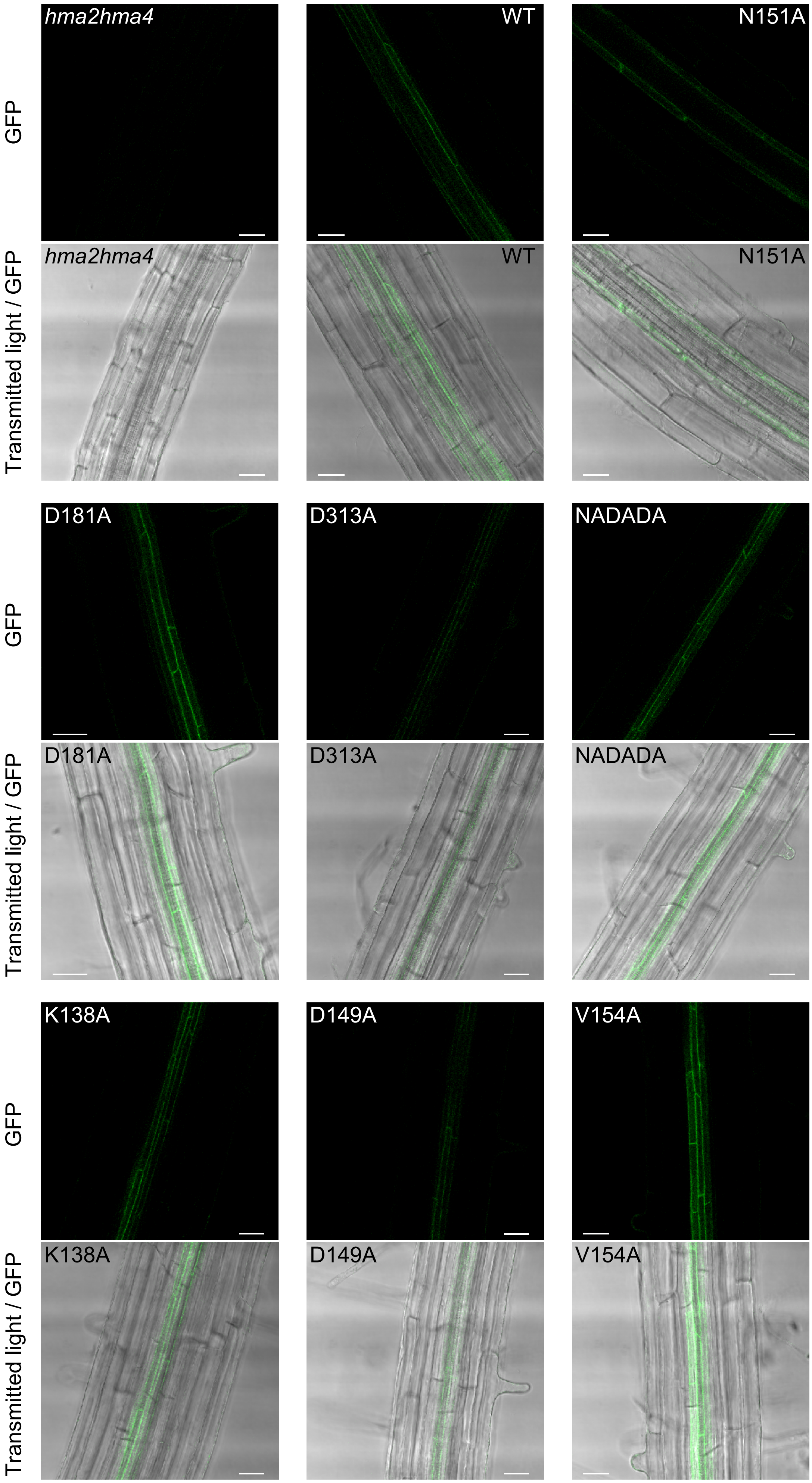

GFP
Transmitted light / GFP
hma2hma4
WT
N151A
D181A
D313A
NADADA
K138A
D149A
V154A

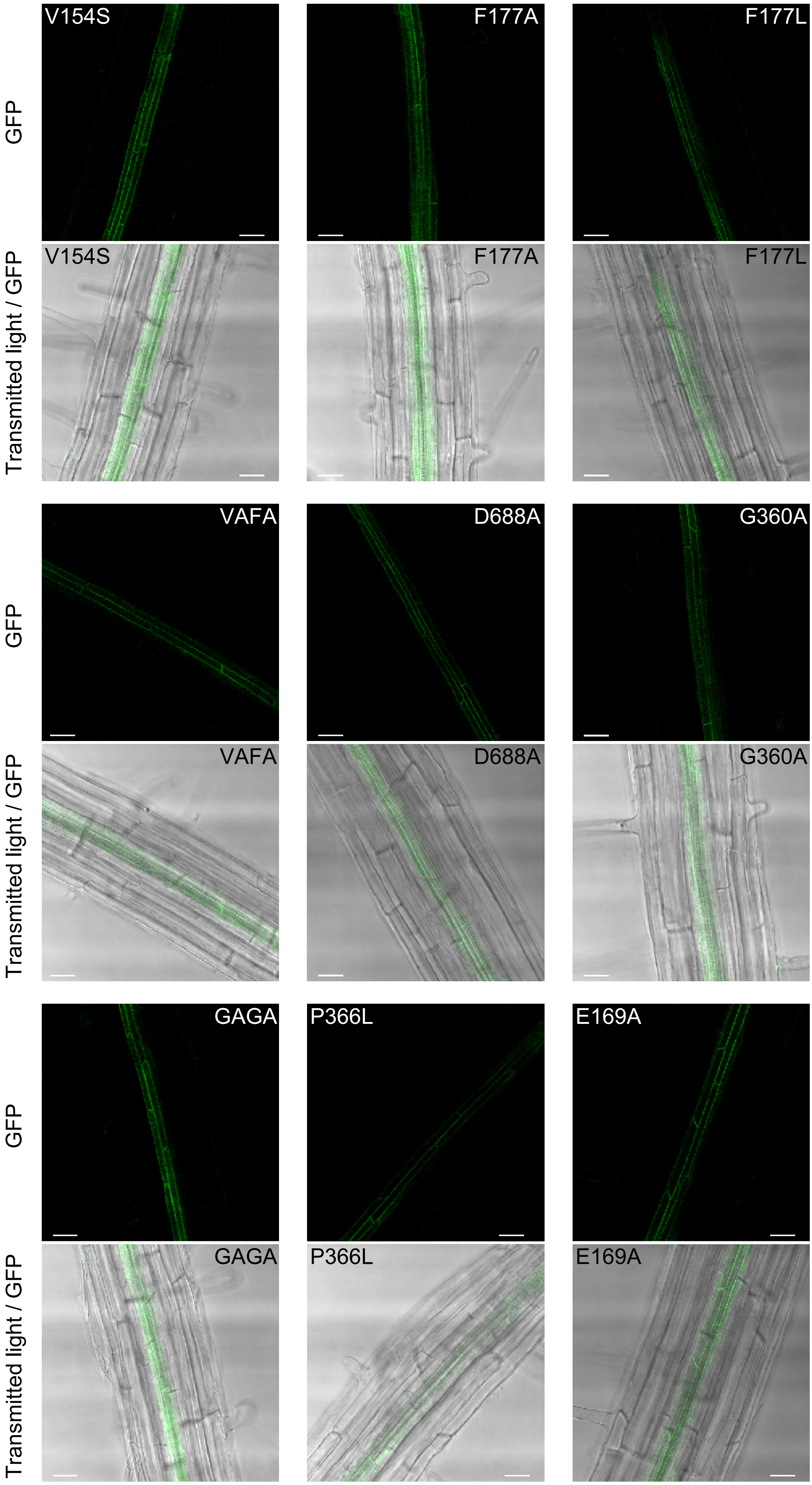
GFP
Transmitted light / GFP
V154S
F177A
F177L
VAFA
D688A
G360A
GAGA
P366L
E169A

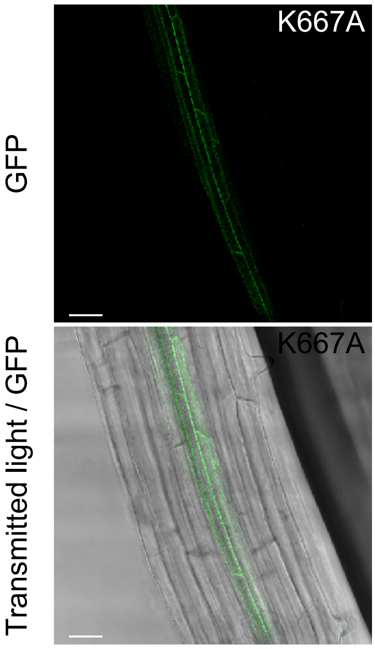

Figure S3. HMA4 TM variant localization in *A. thaliana*. GFP fusions of HMA4 variants were imaged by confocal microscopy in roots of 18 day-old seedlings T1 seedlings. The variants are expressed in the *A. thaliana hma2hma4* mutant under the control of the *pAhHMA4-2* promoter. *hma2hma4* seedlings either non-transformed or expressing the native HMA4 fused to GFP (WT) were respectively used as negative and positive controls. The images are for each variant representative of two to four independent lines from two independent experiments. *Scale bars* 25 µm.

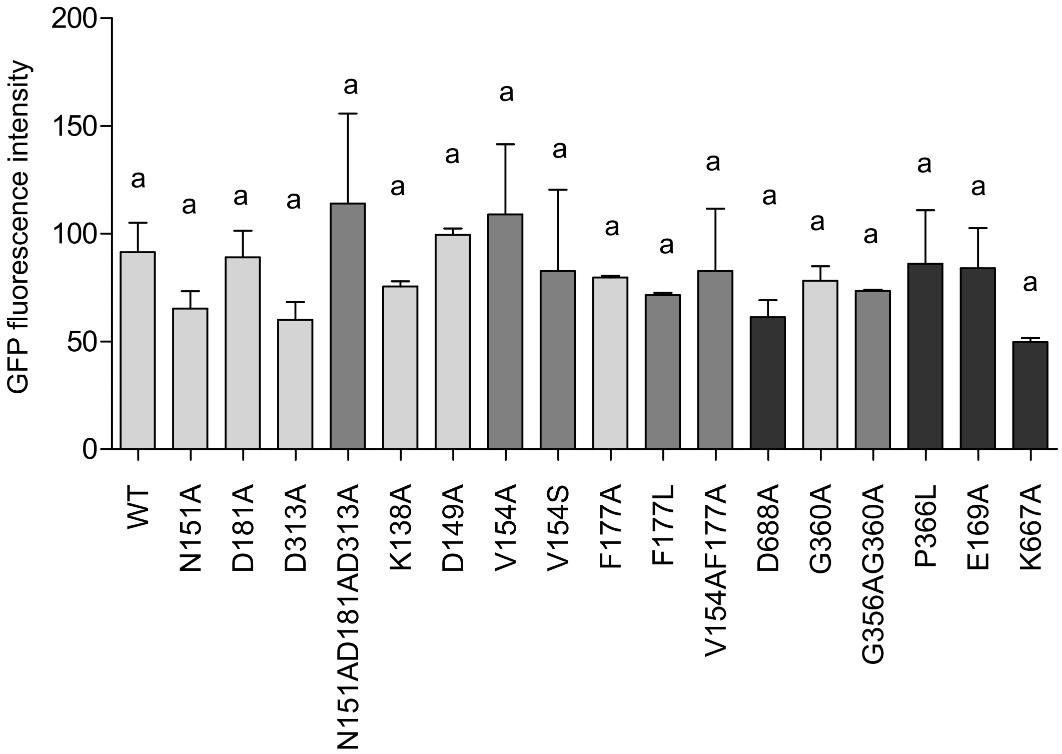

Figure S4. HMA4 TM variant expression level in *A. thaliana*. GFP fusions of HMA4 variants were imaged by confocal microscopy in roots of 18 day-old T1 seedlings. The variants are expressed in the *A. thaliana hma2hma4* mutant under the control of the *pAhHMA4-2* promoter. *hma2hma4* seedlings expressing the native HMA4 fused to GFP (WT) were used as positive controls. Protein expression levels were estimated through quantification of GFP fluorescence in imaged roots. For each genotype, values are mean ± SEM from two to four independent lines from two independent experiments. The data were analyzed with a one-way ANOVA test followed by Tukey's multiple comparison tests. No statistically significant differences were detected. The dark gray, medium gray and light gray colours correspond respectively to plants exhibiting the stunted growth *hma2hma4* phenotype, the WT phenotype but a significant decrease of zinc in shoots and the WT phenotype with a zinc level similar to WT plants (see Fig. 5).

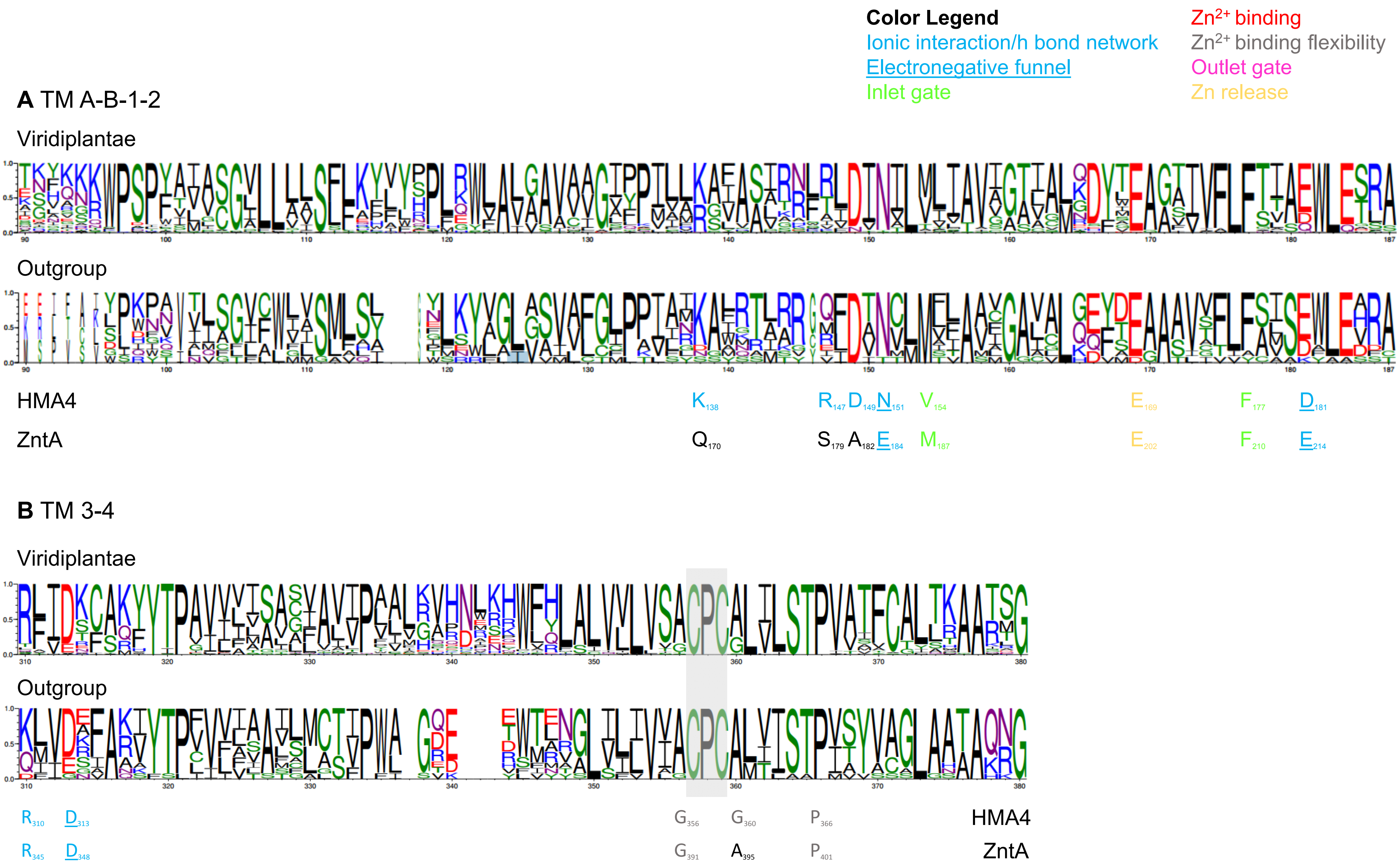

Color Legend
Ionic interaction/h bond network
Electronegative funnel
Inlet gate
Zn2+ binding
Zn2+ binding flexibility
Outlet gate
Zn release
A TM A-B-1-2
Viridiplantae
Outgroup
HMA4
K138 R147 D149 N151 V154 E169 F177 D181
ZntA
Q170 S179 A182 E184 M187 E202 F210 E214
B TM 3-4
Viridiplantae
Outgroup
R310 D313 G356 G360 P366 HMA4
R345 D348 G391 A395 P401 ZntA

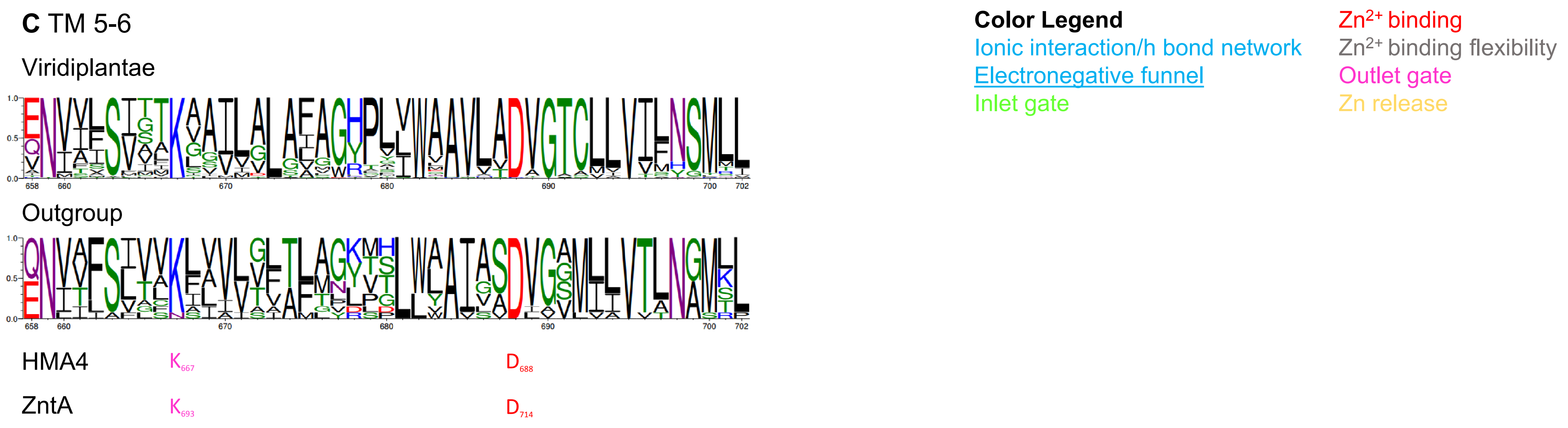

Figure S5. Sequence conservation of the TM region among plant P_{IB-2} ATPases. (A-C) The figure presents sequence logos for TM domains A, B and 1 to 6 of P_{IB-2} ATPases. The height of each letter gives the occurrence frequency of the corresponding amino-acid residue across the aligned sequences (y-axes), without compositional adjustment for simplicity. x-axes are numbered according to the *A. thaliana* HMA4 protein. The logos were computed from a sequence alignment of 124 plant and green algae P_{IB-2} ATPases (Hanikenne and Baurain, 2014). The outgroup corresponds to a small group of P_{IB-2} ATPases found in 13 Stramenopiles. Note that those positions in the alignment that corresponded to gaps in HMA4 were discarded prior generating the logos. The position and function of the HMA4 residues characterized in this study, as well as their state in ZntA are also displayed as follows: the residues forming (i) the ionic interaction/hydrogen bond network are highlighted in cyan, with (ii) the inlet funnel amino acids underlined. The residues involved in (iii) Zn^{2+} binding and (iv) responsible for the binding motif positioning are respectively highlighted in red and gray. The amino acids forming (v) the inlet and (vi) outlet gates are respectively highlighted in green and pink. The residue involved in Zn^{2+} release is highlighted in yellow. The CPC motif, a hallmark of P_{IB-2} ATPases, is on gray background.

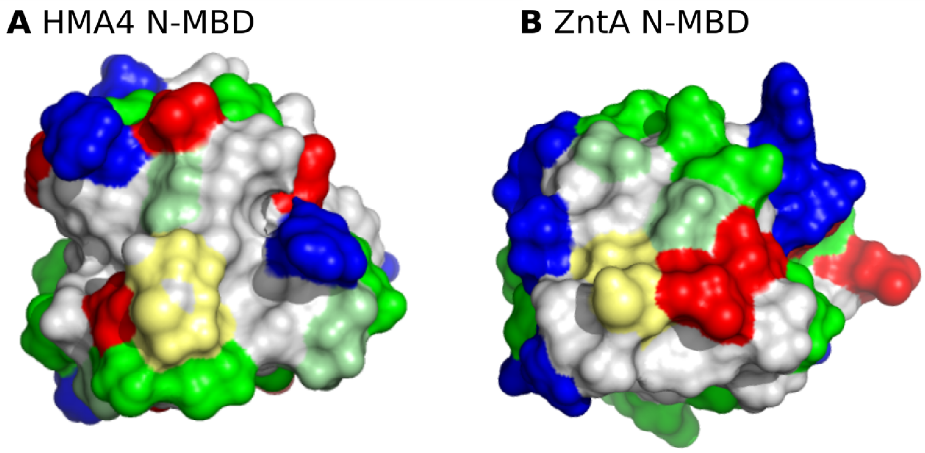

Figure S6. Distribution of residue types at the surface of the N-terminal Metal Binding Domains (MBDs) of *A. thaliana* HMA4 (2KKH) **(A)** and *E. coli* ZntA (1MWY) **(B)**. Hydrophobic, polar, positively charged, negatively charged, cystein and small (Gly and Pro) residues are colored in white, green, blue, red, yellow and pale green, respectively.

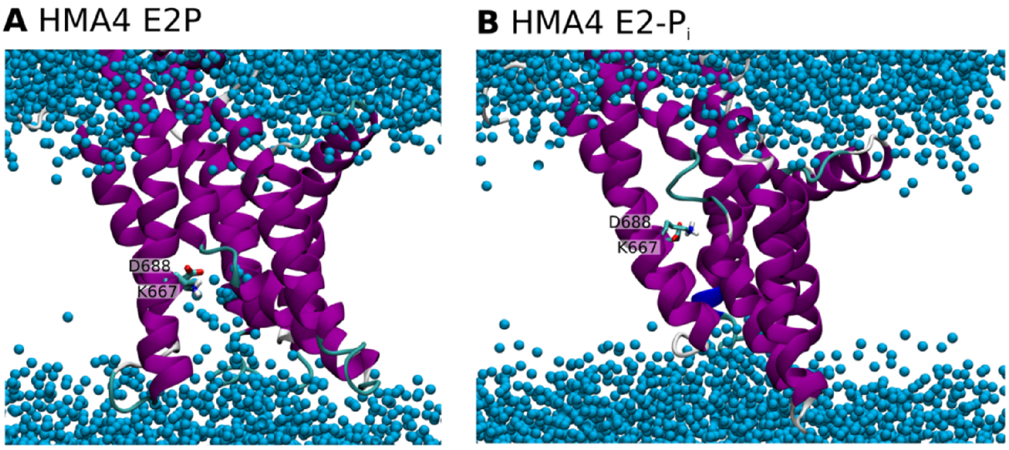

Figure S7. View of the transmembrane domain of HMA4 in the E2P and E2-P_i states in a membrane environment during molecular dynamic simulations. Key residues are in a stick representation and water is represented as blue balls.

```
AtHMA4     FKNKWPSPFAVVSGLLLLLSFLKFVYSPLRWLAVAAVAAGIYPILAKAFASIKRPRIDIN 151
NtHMA4     YQKKWPSPFAIGSGILLGLSFLKYFFAPFQWLALAAVAVGIPPIIFRGVAAVRNLTLDIN 148
OsHMA2     ITNKWPSPYVLLCGLLLVVSLFEHFWHPLKWFALVAAAAGLPPIVLRSIAAIRRLTLDVN 145
OsHMA3     VVSRWPSPYIVASGVLLTASFFEWLFPPLQCLAVAAVVAGAPPMVRRGFAAASRLSLDIN 176

AtHMA4     ILVIITVIATLAMQDFMEAAAVVFLFTISDWLETRASYKATSVMQSLMSLAPQKAIIAET 211
NtHMA4     ILVLIAVAGSIVLHDYWEAGTIVFLFAIAEWLESRASHKATAAMSSLVNIVPPTAVLAES 208
OsHMA2     ILMLIAVAGAIALKDYSEAGFIVFLFTTAEWLETRASHKATAGMSALMSMAPQKAILAET 205
OsHMA3     VLMLIAVAGALCLGDYTEAGAIVFLFTTAEWLETLACTKASAGMSSLMGMLPVKAVIATT 236

AtHMA4     GEEVEVDEVKVDTVVAVKAGETIPIDGIVVDGNCEVDEKTLTGEAFPVPKQRDSTVWAGT 271
NtHMA4     GEVVNVDEVKVNSILAVKAGETIPIDGVVVEGECDVDEKTLTGESFPVSKQRDSTVWAGT 268
OsHMA2     GEVVAARDVKVNTVIAVKAGEVIPIDGVVVDGRSEVDESTLTGESFPVSKQPDSQVWAGT 265
OsHMA3     GEVVSVRDVRVGDVVAVRAGEIVPVDGVVVDGQSEVDERSLTGESFPVPKQPHSEVWAGT 296

AtHMA4     INLNGYICVKTTSLAGDCVVAKMAKLVEEAQSSKTKSQRLIDKCSQYYTPAIILVSACVA 331
NtHMA4     TNLNGYISVKTTALAEDCAVARMAQLVEDAQNKKSKTQRYIDKCAKYYTPAIVAISASLA 328
OsHMA2     LNIDGYIAVRTTAMADNSAVAKMARLVEEAQNSRSSTQRLIDTCAKYYTPAVVVMAGSVA 325
OsHMA3     MNFDGYIAVRTTALAENSTVAKMERLVEAAQNSRSKTQRLIDSCAKYYTPAVVVVAAGVA 356

AtHMA4     IVPVIMKVHNLKHWFHLALVVLVSGCPCGLILSTPVATFCALTKAATSGLLIKSADYLDT 391
NtHMA4     IVPTALRVHNRNEWYRLALVTLVSACPCALVLSTPVAMCCALSKAATSGLLFKGAEYLET 388
OsHMA2     AIPAIAKAHNLKHWFQLALVLLVSACPCALVLSTPIATFCALLRAARTGLLIKGGDVLES 385
OsHMA3     LIPALLGADGLEQWWKLALVMLVSACPCALVLSTPVASFCAMLRAARMGIFIKGGDVLES 416

AtHMA4     LSKIKIVAFDKTGTITRGEFIVIDFKSLSR-DINLRSLLYWVSSVESKSSHPMAATIVDY 450
NtHMA4     LAKIKIMAFDKTGTITKGEFMVTEFKSLID-GFSLNTLLYWVSSIESKSGHPMAAALVDY 447
OsHMA2     LASIKVAAFDKTGTITRGEFSVEEFQPVGE-RVSLQQLLYWVSSVESRSSHPMASVLVDY 444
OsHMA3     LGEIRAVAFDKTGTITRGEFSIDSFHLVGDHKVEMDHLLYWIASIESKSSHPMAAALVEY 476

AtHMA4     AKSVSVEPRPEEVEDYQNFPGEGIYGKIDGNDIFIGNKKIASRAGCSTVPEIEVDTKGGK 510
NtHMA4     AQSNSVEPKPDRVEQFQNFPGEGIFGRIDGMEIYVGNRKISSRAGCTTVPEIEGDSFKGK 507
OsHMA2     AQSKSVEPKSENVSEFQIYPGEGIYGEIDGAGIYIGNKRILSRASCETVPDMK--DMKGV 502
OsHMA3     AQSKSIQPNPENVGDFRIYPGEGIYGEIHGKHIYIGNRRTLARASSPQSTQEMGEMIKGV 536

AtHMA4     TVGYVYVGERLAGFFNLSDACRSGVSQAMAELKSLGIKTAMLTGDNQAAAMHAQEQLGNV 570
NtHMA4     SVGYIFLGSSPAGIFSLSDVCRIGVKEAMRELKQMGIKTAMLTGDCYAAANHVQDQLGGA 567
OsHMA2     TIGYVACNNELIGVFTLSDACRTGSAEAIKELRSLGIKSVMLTGDSSAAATYAQNQLGNI 562
OsHMA3     SIGYVICDGELAGVFSLSDDCRTGAAEAIRELGSLGIKSVMLTGDSSAAATHAQGQLGGV 596

AtHMA4     LDVVHGDLLPEDKSRIIQEFK-KEGPTAMVGDGVNDAPALATADIGISMGISGSALATQT 629
NtHMA4     LDEFQAELLPEDKATIIKGFQ-KEAPTAMIGDGLNDAPALATADIGISMGISGSALAKET 626
OsHMA2     LAEVHAELLPEDKVRIVGELKEKDGPTLMVGDGMNDAPALAKADVGVSMGVSGSAVAMET 622
OsHMA3     MEELHSELLPEDKVRLVSGLKARFGPTMMVGDGMNDAAALAAADVGVSMGISGSAAAMET 656

AtHMA4     GNIILMSNDIRRIPQAVKLARRARRKVVENVCLSIILKAGILALAFAGHPLIWAAVLVDV 689
NtHMA4     GHVILMTNDIGRIPKAARLARRVRRKIVENMIISVVTKAAIVALAIAGYPLVWAAVLADT 686
OsHMA2     SHVALMSNDIRRIPKAVRLARRTHRTIIVNIIFSVITKLAIVGLAFAGHPLIWAAVLADV 682
OsHMA3     SHATLMSSDVLRVPEAVRLGRCARRTIAVNVAGSVAVKAAVLALAAAWRPVLWAAVLADV 716

AtHMA4     GTCLLVIFNSMLLLREK 706
NtHMA4     GTCLLVILNSMLLLRGG 703
OsHMA2     GTCLLVIMYSMLLLREK 699
OsHMA3     GTCLLVVLNSMTLLREE 733
```

Figure S8. Amino acid sequence alignment of *Arabidopsis thaliana* HMA4, *Nicotiana tabacum* HMA4, *Oryza sativa* HMA2 and *Oryza sativa* HMA3. The AtHMA4 amino acids depicted by the 3D model as important for Zn^{2+} transport and conserved in NtHMA4, OsHMA2 and OsHMA3 are

highlighted. The alignment was obtained using Clustal Omega with default settings (Sievers et *al*., 2011. Molecular systems biology **7**, 539).

Table S1. Mutagenic primers.

Name	Sequence (5'=>3')
AtHMA4 N151A	ggcctaggatcgacatc**gc**catattggtcataataacc
AtHMA4 D181A	tattcaccatatccg**c**ctggctcgaaacaagagc
AtHMA4 D313A	tctcagagactaatag**c**caaatgttctcagtac
AtHMA4 K138A	ctatccgattcttgcc**gc**agcctttgcttcc
AtHMA4 D149A	ccattaaaaggcctaggatcg**c**catcaacatattgg
AtHMA4 V154A	cgacatcaacatattgg**c**cataataaccgtgattgc
AtHMA4 V154S	ggatcgacatcaacatattg**tc**cataataaccgtgattgc
AtHMA4 F177A	gcagttgtgttccta**gc**caccatatccgactgg
AtHMA4 F177L	gcagttgtgttccta**c**tcaccatatccgactgg
AtHMA4 D688A	gggctgcggttcttgttg**c**tgtagggacttgtctg
AtHMA4 P366L	gtcttatcctctctacac**t**agttgctactttctgtgc
AtHMA4 G360A	tggttgtccctgtg**c**tcttatcctctctacacc
AtHMA4 G356AG360A	gtgttagtcagtg**c**ttgtccctgtg**c**tcttatcctctc
AtHMA4 E169A	gcaagatttcatgg**c**ggcagcagcagttgtg
AtHMA4 K667A	gtctatcgatcattta**gc**agcaggaatactcgct

Mutated bases are indicated in underlined bold font.

Table S2. RMSD (in nm) after 25 ns of molecular dynamic simulations for the membrane domain of the native HMA4 protein (WT) and its mutants in the E2P and E2-P_i states.

HMA4 mutants	E2P	E2-P_i
WT	0.27	0.24
N151A	0.19	0.17
N151A - D181A - D313A	0.24	0.17
D181A	0.26	0.21
D313A	0.20	0.19
K138A	0.24	0.20
D149A	0.27	0.19
V154A	0.27	0.23
V154A - F177A	0.28	0.26
V154S	0.26	0.21
F177A	0.23	0.22
F177L	0.25	0.17
D688A	0.26	0.20
G356A - G360A	0.26	0.23
G360A	0.30	0.19
P366L	0.36	0.22
E169A	0.22	0.19
K667A	0.25	0.21
